# One-step Multiplex Transgenesis via *Sleeping Beauty* Transposition in Cattle

**DOI:** 10.1038/srep21953

**Published:** 2016-02-24

**Authors:** Wiebke Garrels, Thirumala R. Talluri, Ronja Apfelbaum, Yanet P. Carratalá, Pablo Bosch, Kerstin Pötzsch, Esther Grueso, Zoltán Ivics, Wilfried A. Kues

**Affiliations:** 1 Friedrich-Loeffler-Institut, Institut für Nutztiergenetik, Neustadt, Germany; 2 Institute for Laboratory Animal Sciences, Medical School Hannover (MHH), Germany; 3 Animal Biotechnology Department, Center for Genetic Engineering and Biotechnology, Havana, Cuba; 4 Departamento de Biología Molecular, FCEFQyN, Universidad Nacional de Río Cuarto, Córdoba, Argentina; 5 Paul-Ehrlich-Institute, Langen, Germany

## Abstract

Genetically modified cattle are important for developing new biomedical models and for an improved understanding of the pathophysiology of zoonotic diseases. However, genome editing and genetic engineering based on somatic cell nuclear transfer suffer from a low overall efficiency. Here, we established a highly efficient one-step multiplex gene transfer system into the bovine genome.

Genetically modified cattle are important for developing new biomedical models^1^,^2^, and for an improved understanding of the pathophysiology of zoonotic diseases, like tuberculosis and bovine spongiform encephalopathy (BSE)^3^,^4^. Recently, it was demonstrated that designer nucleases, such as zinc finger nucleases (ZFN) and transcription activator-like effector nucleases (TALEN) are suitable for genome editing in cattle^2^,^5^,^6^,^7^. However, genome editing and genetic engineering based on somatic cell nuclear transfer (SCNT) suffer from a low overall efficiency^1^,^2^,^3^,^4^, due to extensive micromanipulation and nuclear reprogramming failures. The SCNT protocol includes sequential micromanipulation steps (enucleation, transfer of donor cell, fusion of cytoblast and donor cell, and activation of reconstructed embryos), which requires highly skilled experimenters, reliable oocyte supply and a large herd of surrogate animals. In addition, animal cloning is associated with increased rates of abortion and health problems, such as the large offspring syndrome, due to incomplete epigenetic reprogramming of the somatic donor nuclei^8^. Considering the long gestation (9 months) and long generation interval (22–26 months) in cattle, more efficient methods are required to fully exploit the biotechnological potential and to increase the pace of genetic engineering in this species.

Here, we established a highly efficient one-step gene transfer system into the bovine genome of *in vitro* fertilized (IVF) zygotes, allowing the simultaneous integration of several independent transgenes delivered by the *Sleeping Beauty* (SB) transposon system.

## Results

**Figure 1:**
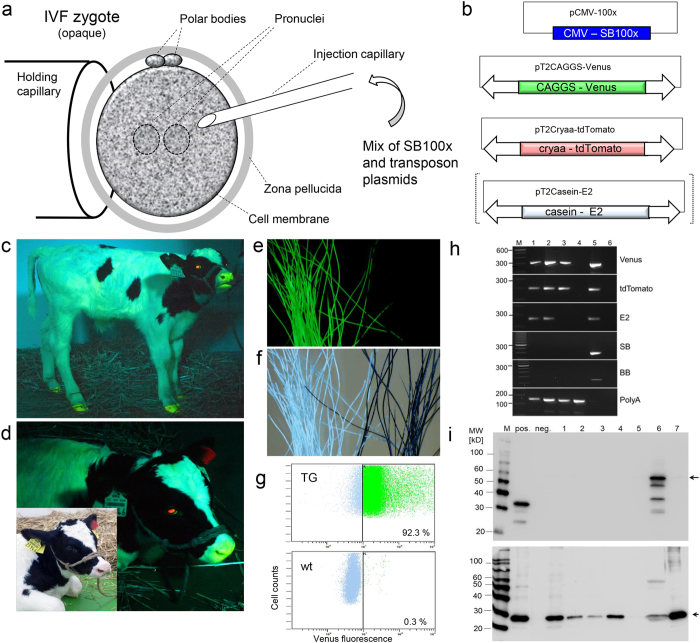
One-step generation of multi-transgenic cattle. (**a**) Cytoplasmic injection of transposon plasmids into bovine zygotes. Mixtures of the helper plasmid, encoding the hyperactive SB100× transposase and either two (pT2CAGGS-Venus and pT2Cryaa-tdTomato) or three transposons (pT2CAGGS-Venus, pT2Cryaa-tdTomato and pT2Casein-E2) were co-injected into the opaque cytoplasm of a bovine zygote. Upon expression of the SB100× transposase, the enzyme will catalyze integration of the transposon constructs by a precise cut-and-paste mechanism^13^,^23^. (**b**) Schematic depiction of the used plasmids. pCMV-SB100×, expression plasmid of transposase (helper plasmid); and three SB transposon plasmids: pT2CAGGS-Venus, ubiquitously active CAGGS promoter driving Venus fluorophore; pT2Cryaa-tdTomato, lens specific promoter driving tdTomato; pT2Casein-E2, udder-specific promoter driving E2 subunit of classical swine fever virus. Arrows indicate SB inverted terminal repeats. (**c**) Triple transposon transgenic calf shown under excitation of Venus and tdTomato. Note the widespread Venus-fluorescence in snout, hooves and hair; and the lens-specific expression of tdTomato. (**d**) Same animal as in C). Inset, imaged under white light conditions. (**e**) Expression of the Venus fluorophore in white hair, in black hair the fluorescence is quenched. Wildtype controls are shown in Fig. S2. (**f**) Corresponding control image to E) (white light condition). (**g**) Expression of Venus in leukocytes from triple-transgenic calf (TG) and a wildtype control (wt). (**h**) Genotyping of transgenic calves by PCR. M, size marker; 1, 2 triple transgenic calf; 3, double-transgenic calf; 4, wt gDNA; 5, positive control (plasmids, except for PolyA PCR); 6, no template control. Venus, tdTomato and E2 PCRs indicate presence of the respective transposon; SB and BB PCRs indicate absence of helper plasmid and transposon backbones; PolyA, positive control PCR. (**i**) Immunodetection of lens-specific expression of tdTomato (top) and ubiquitous expression of Venus (bottom) in organ samples from the double-transgenic calf by Western blotting. M, molecular size marker (Magic mark); pos., positive control (mCherry and Venus); neg., negative control (wildtype muscle sample); 1, muscle; 2, liver; 3, kidney; 4, heart; 5, vitreous body; 6, eye lens; 7, fat tissue. Arrows indicate calculated the molecular weight of tdTomato (54 kD) and Venus (29 kD).

Modular mixtures of the transposons and a helper plasmid, encoding the hyperactive SB100× transposase were co-injected into the cytoplasm of IVF zygotes (Fig. 1; Table 1). Upon expression of the SB100× transposase, the enzyme catalyzes independent integrations of the transposon constructs into the bovine genome.

Bovine zygotes were produced from oocytes derived from slaughterhouse ovaries by *in vitro* maturation and IVF with frozen-thawed sperm according to standard protocols on two experimental days. Altogether 134 zygotes were injected with a plasmid mixture of pCVM-SB100×, and either two (pT2CAGGS-Venus and pT2Cryaa-tdTomato) or three transposons (pT2CAGGS-Venus, pT2Cryaa-tdTomato and pT2Casein-E2) at 20–24 hours post-fertilization (Table 1).

The pCAGGS-Venus transposon carried a ubiquitously active promoter (CAGGS) driving the Venus fluorophore. The pCryaa-tdTomato transposon carried a lens cell-specific crystallin A alpha promoter driving the tandem (td) Tomato fluorophore, and the pCasein-E2 transposon carried an udder-specific promoter driving a recombinant E2 glycoprotein of classical swine fever virus. The Venus and tdTomato reporter constructs allowed for the vital recording of widespread or cell type-restricted expression (Fig. 1), respectively. An approximate volume of 10 pl with a DNA concentration of 30 ng/μl and equimolar quantities of the plasmids was injected directly into the cytoplasm (Fig. 1a,b), without attempting to identify and to inject a pronucleus. This “blind” deposition into the cytoplasm allows a more rapid pace of the injection procedure, and avoids any high-speed centrifugation to reveal the pronuclei in the opaque bovine zygotes. Injected (n = 134) and control (n = 155) zygotes were cultured in synthetic oviduct fluid (SOF) in a humidified atmosphere with reduced oxygen (5% O_2_). After 7 days of culture, the embryos were inspected for development to blastocysts and expression of the Venus reporter. The injections on the two experimental days were performed by two different experimenters, respectively, which beside the variable quality of the abattoir ovaries may have contributed to the different developmental rates to blastocysts (22.2 and 11.2% in the treatment groups). Venus-positive blastocysts were selected for embryo transfer (ET, 1 blastocyst was transferred per recipient) to synchronized surrogate animals. A total of 9 blastocysts at day 8 of *in vitro* culture with reporter expression of Venus were obtained. Due to scarcity of recipient animals only 6 blastocysts were used for embryo transfer. In the injection groups, reduced rates of blastocyst formation were found compared to the culture controls, which may indicate that the treatment reduced the regular cleavage divisions. On experimental day 1 two blastocysts, and on experimental day 2 four blastocysts were transferred to synchronized surrogates (n = 6), resulting in two pregnancies (Table 1). Both pregnancies run to term, and two normally developed and vital calves, a male and a female were delivered by Cesarean section.

The female calf was normal sized, was normally breathing and did not show any visible abnormalities, unfortunately the initial veterinarian assessment did not detect an aneurism directly at the connection of the umbilical cord to the peritoneum. After 15–20 minutes, when the calf attempted to stand up, the aneurism was ruptured, leading rapidly to a hypovolemic shock and death. Also, the necropsy did not reveal any macroscopic abnormalities, except the aneurism (Supplementary material, Fig. S1). The male calf (Fig. 1c–g) developed normally and is currently 8 months old. Phenotypic analysis suggested that both calves were indeed multi-transgenic, showing widespread expression of Venus and lens-specific expression of tdTomato (Fig. 1, Supplemental Fig. S1), this was confirmed by immunoblotting of samples from the deceased female calf (Fig. 1i). Genotyping and molecular analyses confirmed the integration of both reporter transposons in the female calf (Table 1; Supplemental Table S1). The male calf was confirmed to be triple-transgenic, carrying all three transposons. The identification of flanking genomic sequences by splinkerette PCR identified individual SB transposase-catalyzed integrations in consensus TA-dinucleotide sequences (Supplemental Table S1), on chromosomes 6, 7, 10, 11, within a repetitive element, and two positions, which could not be annotated in the bovine genome due too short flanking sequences. The absence of helper plasmid and transposon backbone sequences in the genomes of the transgenic calves was confirmed by PCR (Fig. 1h). Deposition of the ubiquitously expressed Venus in the hair was detected (Fig. 1e,f; Supplementary Fig. S2).

Flow cytometric analysis of primary fibroblasts derived from the female calf showed that 95% of the cells were Venus positive, whereas 88% of fibroblasts and 92–95% of leukocytes from the male calf were Venus positive, suggesting that the founders showed some cellular mosaicism, which may be due to integration after the one-cell stage (Supplementary Fig. S3–S5).

## Discussion

The here established cytoplasmic injection of zygotes with SB transposon plasmids is both technically simple and highly efficient for multiplex transgenesis in cattle. Pre-selection of Venus-positive bovine blastocysts for embryo transfer after one week of *in vitro* culture likely contributed to the 100% rate of multi-transgenic calves per born calves. The SB catalyzed gene transfer resulted in precise transposition of the reporter cassettes flanked by the ITRs of the SB transposon. This is the first description of multi-transgenic cattle generated by a microinjection technique, allowing for the generation of complex transgenic genotypes in the cattle model within a reasonable time frame.

In the first experiments striving to generate transgenic cattle by standard pronuclear microinjection, the experimenters typically injected thousands of zygotes^9^,^10^,^11^ and performed dozens of embryos transfers, resulting at best in about 10% transgenic calves per born animals. Even worse, only a sub-fraction of the transgenic cattle showed expression of the transgene, thus further reducing the ratio of “useful” transgenic offspring. Here, we obtained two multi-transgenic calves out of two born calves from a limited number of injected zygotes (n = 134). The main factors reducing the efficiency of the here described transgenic approach are the current methods for *in vitro* culture for bovine embryos and embryo transfer of IVF-derived blastocysts to synchronized surrogate animals. Standard (commercial) IVF systems commonly achieve blastocyst rates of no more than 30% of the fertilized zygotes, and embryo transfer of IVF-derived blastocysts typically result in pregnancy rates of no more than 50% after a single embryo transfer. Considering these general limitations and the availability of only 6 surrogate animals, the overall success rate of two multi-transgenic calves out of 134 treated IVF zygotes compares rather favorable to previous approaches.

This approach expands the toolbox of genome engineering technologies in cattle, it is likely that experiments striving for co-expression, conditional expression or gene knock-down in cattle will benefit from the strategy described here. A further advantage of the transposition-based gene delivery strategy is that no unwanted DNA sequences, such as antibiotic markers or plasmid backbones, will be introduced into the genome^12^. Importantly, the transposon-based approach is simple, efficient and allows the modular design of genetic modifications.

Both calves showed some cellular mosaicism for the Venus transposon driven by the ubiquitously active CAGGS promoter, resulting in 5–12% Venus-negative cells. The Venus-negative cells did not carry the reporter transposon, as determined by flow cytometric sorting and PCR amplification (Fig. S5). The cellular mosaicism may be due to transposition after the one-cell stage, or alternatively due to re-mobilization of an early integration event without a secondary integration in some blastomeres, depending on the half-life and distribution of the transposase enzyme. Previous own experiments with Sleeping Beauty transposition in murine and porcine zygotes, however, suggest that this low level of cellular mosaicism in the founders is not critically for germline transmission of the introduced DNA transposons^13^,^14^.

The one-step in ovo transposition might be of particular interest for genetic approaches, where two or more transgenes need to be inserted into the bovine genome. It has been shown that the expression of recombinant proteins in the udder can be enhanced by co-expression of enzymes for posttranslational modification^15^. The co-expression of recombinant proteins, which have synergistic effects^16^,^17^ or different subunits, which assemble to functional protein complexes^18^,^19^ would be simplified by this method. The stable integration of binary cassettes of inducible systems or designer nuclease components, which may confer disease resistance, are other possible application scenarios^20^.

## Material and Methods

### Ethics statement

Animals were maintained and handled according to the German laws regulating animal welfare, and genetically modified organisms. The experiments were approved by an external ethics committee (Niedersächsisches Landesamt für Verbraucherschutz und Lebensmittelsicherheit, AZ 33.19-42502-04-12/0777).

### Production of bovine *in vitro* zygotes

Ovaries were collected from a commercial slaughterhouse, and cumulus-oocyte-complexes (COC) were released by slicing with a blade. The COC were matured in TCM medium for 24 hours, COCs with expanded cumulus layer were then fertilized *in vitro*, and presumptive fertilized oocytes were placed in TCMair medium^21^.

### Sleeping Beauty transposon system plasmids

The pCMV-100x and pT2CAGGS-Venus plasmids were described before^13^. For the pT2Cryaa-tdTomato transposon, the Cryaa promoter-tdTomato gifted by Dr. Xu (Yale)^22^ was subcloned between the SB ITRs. In the pT2Casein-E2 transposon, a bovine/buffalo casein promoter-E2 subunit cDNA of classical swine fever was subcloned between the SB ITRs. The plasmids were amplified in XL10 or DH5alpha E. coli strains and purified by commercial anion exchange columns (Qiagen Tip 100).

### Cytoplasmic injection of transposon plasmids

The transgenic calves were generated by cytoplasmic plasmid injection of SB plasmid mixtures into bovine IVF zygotes essentially as described previously for porcine zygotes^13^,^23^. In brief, equimolar mixtures of the plasmids with a total DNA concentration of 30 ng/μl in injection buffer (10 mM TrisHCl pH 7.4/ 0.25 mM EDTA) were prepared directly before injection. The injection solution was backfilled in injection capillaries with a filament. Injection and holding capillaries were arranged on micromanipulators on a micromanipulation unit with an inverted microscope^23^.

Small groups of 5–10 IVF zygotes were transferred into a drop of medium (500 μl) on a glass plate. Unlike for pronuclear injection, here no high speed centrifugation with ~15 000 g is required. The non-centrifuged zygotes appear opaque, and shall bear two polar bodies, however the pronuclei were not discernable.

Individual zygotes were fixed on the holding capillary by gentle suction, and then the Zona pellucida and the cell membrane were penetrated with the injection capillary. Approximatly 10 picoliter (pl) of the plasmid injection was deposited inside the cytoplasm.

### *In vitro* culture of injected embryos

Treated zygotes were then put in a 30 μl drop of synthetic oviduct fluid (SOF) covered with mineral oil and incubated at 39 °C in an atmosphere with reduced oxygen (5%) for 8 days. Then the number of developed blastocysts was determined, and the expression of Venus was checked under a microscope with epifluorescence.

### Embryo transfer and determination of pregnancy

Venus-positive blastocysts were individually loaded in embryo transfer straws (Minitube Minipalilette 0.25 ml) in 100- μl of PBS. Non-surgical ET was performed on hormonally synchronized surrogate animals. The establishment of a pregnancy was tested by palpation and ultrasound analysis 30 days later.

### Fluorescence microscopy and macroscopic fluorescence excitation of animals

For fluorescence microscopy of cell cultures, a Zeiss Axiovert 35M microscope equipped with fluorescence optics was used. For specific excitation of Venus a filter block with excitation of 450–490 nm and emission of 520–550 nm, for specific excitation of tdTomato a filter block with excitation of 530–570 nm and emission of 590–610 nm were used.

For imaging of tissue biopsies an Olympus SZ16 stereomicroscope with epifluorescence optics was used. For macroscopic imaging of the calves, either blue or green flood lights of light emitting diodes (LED; Eurolite) were used for excitation, and images were recorded with a digital camera and appropriate emission filters (Lee Filter)^14^.

### Genotyping and identification of integration sites

Transposon-genomic DNA junctions were determined using splinkerette PCR as described^24^. The purified PCR product was cloned into the pGEM-Teasy vector (Promega, Madison, USA), and the DNA sequence was determined by standard sequencing technology (ABI3730XL Applied Biosystems, Foster City, California). Sequences were analyzed with BLAST (www.ensembl.org). Southern blots and PCR reactions of genomic DNA were done according to standard procedures^13^,^23^.

### Preparation of primary cell cultures and FACS measurements

Primary cells were derived from tissue samples of newborn calves as described^13^ and cultured in DMEM supplemented with 10% fetal calf serum, 2 mM L-glutamine and antibiotics. Leukocytes were isolated from EDTA-blood samples and re-suspended in PBS. Flow cytometry analysis of primary cells and leukocytes was performed using a FACScan (BD Bioscience, Heidelberg, Germany). Samples were diluted to 0.5 × 10^6^ cells/ml and measured in duplicates, acquiring 10 000 cells per sample. Cells with membrane damage were excluded from the analysis by counterstaining with propidium iodide (20 μM).

### Western blotting

Finely grinded tissues and cells were extracted in RIPA buffer, and 10 microgram of protein per slot was separated on a 10% SDS-PAGE gel, blotted to a PVDF membrane, blocked in 5% non-fat milk powder and probed with rabbit polyclonal antibodies against EGFP (Thermo) or mCherry (Santa Cruz) in 1:1000 dilutions, respectively, followed by a secondary anti-rabbit antibody in 1:10 000 dilution (Sigma-Aldrich). The anti-mCherry antibody cross-reacts with other red fluorescent proteins, and is suitable for the detection of tdTomato (calculated molecular weight 54 kD). For chemiluminescence detection an ECL + kit (GE Healthcare) and an image acquisition system (Vilber Lourmat, Fusion SL 3500) were used. As positive control for Western blotting a double transgenic porcine muscle tissue, expressing both Venus and mCherry, was used.

## Additional Information

**How to cite this article**: Garrels, W. *et al.* One-step Multiplex Transgenesis via *Sleeping Beauty* Transposition in Cattle. *Sci. Rep.* **6**, 21953; doi: 10.1038/srep21953 (2016).

## Supplementary Material

Supplementary Information

## Figures and Tables

**Table 1:** One-step multiplex transgenesis in cattle.

Experi-mental day	Treatment	No of zygotes	Injection mixture	No of blastocysts, day 8 (%)	No of Venus-positive blastocysts	No of ETs	No of pregnancies (day 30)	No of calves	No of transgenic calves (% of ET/ % of born)	Sex/birth weight
1	CPI	45	pCMV-SB100×, pT2CAGGS-Venus, pT2Cryaa-tdTomato.	10 (22.2)	5	2	1	1	1, double transgenic (50/ 100)	Female/42 kg
	Culture control	50	n.a.	19 (38.0)	0	0	n.a.	n.a.	n.a.	n.a.
2	CPI	89	pCMV-SB100×, pT2CAGGS-Venus, pT2Cryaa-tdTomato, pT2Casein-E2.	10 (11.2)	4	4	1	1	1, triple transgenic (25/100)	Male/45 kg
	Culture control	105	n.a.	18 (17.1)	0	0	n.a.	n.a.	n.a.	n.a.

n.a., not applicable.
