# Supplementary material for: One-step Multiplex Transgenesis via Sleeping Beauty Transposition in Cattle
Source: Sci Rep. 2016 Feb 24;6:21953. doi: 10.1038/srep21953 (PMC4764937; doi:10.1038/srep21953)
Supplement: Supplementary Information [file srep21953-s1.pdf]

Supplementary Information

One-step Multiplex Transgenesis via *Sleeping Beauty* Transposition in Cattle

Wiebke Garrels[1,2,§], Thirumala R. Talluri[1,§,#], Ronja Apfelbaum[1], Yanet P. Carratalá[1,3], Pablo Bosch[4], Kerstin Pötzsch[5], Esther Grueso[5], Zoltán Ivics[5], Wilfried A. Kues[1]

[1]Friedrich-Loeffler-Institut, Institut für Nutztiergenetik, Neustadt, Germany;

[2]Institute for Laboratory Animal Sciences, Medical School Hannover (MHH), Germany;

[3]Animal Biotechnology Department, Center for Genetic Engineering and Biotechnology, Havana, Cuba;

[4]Departamento de Biología Molecular, FCEFQyN, Universidad Nacional de Río Cuarto, Córdoba, Argentina;

[5]Paul-Ehrlich-Institute, Langen, Germany.

Tab.S1 Integration site analysis in SB transposon calves

Calf	Genomic Sequence	Genomic location	Identity
**female**			
A	TTCCTGAAGTTGCATTGAAGAATTTTAAAGCGTTTTGCTAATAATTCCTACTCTTCCAAATA	**Chr 6** alt assembly Btau 4.6.1 26 994 564	100.0 %
B	GCGAGATGGTGGTGGTGGGGGACAGCCCCCAGTGAAATCACAGGTGAGAGCATAAAGCAATA	**1.711a satellite DNA** (repetitive element)	95.7 %
**male**			
1.1	TTTAAGTCACTGAGTTTGTGGTAGTTTGTTATTGCAGCTTCAGGAGGCTAACATAAGACATA	**Chr 10,** 1 854 048	100.0 %
2.1	TACCAAGTATTACTTGATCTGAATTTCAGTAATGATTTATGATTCTTAAGCCTTTGATACTA	**Chr 11,** 34 661 774	100.0 %
1.5	AATATCTCCTGGAGAAGGAAATGGCAACCCACTCCAGTATTCTTGCCTGGAGAATCCCTATA	**Chr 6,** 95 932 721, intergenic	98.9 %
1.12	GGTAATTTAAGCCCTATGAACACTTAATGNAGCAGGAAATATATATTTGTGTGTGTAATTTA	**Chr 7,** 779 939 305, intergenic	98.9 %
4.1	CCCTATA	too short	-
5.1	AGATA	to short	-

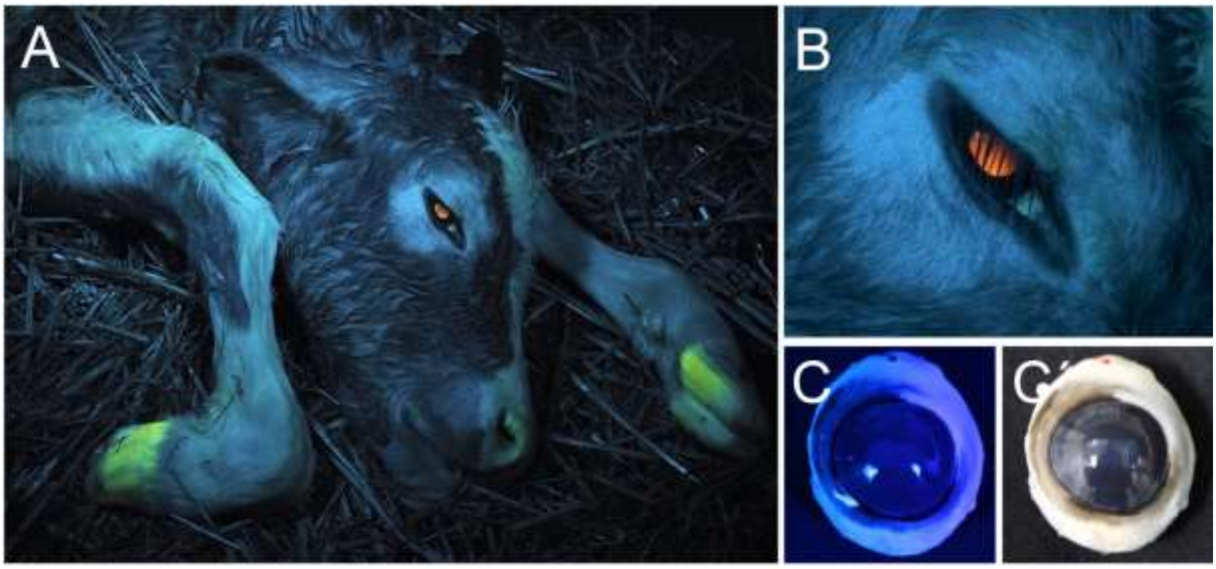

Fig. S1 Phenotype of double-transgenic female calf

A) Newborn calf imaged under specific excitation of Venus and tdTomato. Note the widespread expression of Venus particularly in snout and hoves. The hair contain also Venus, however the hair act as optical fibers and scatter the fluorescence light, in the black hair the fluorescence is quenched (see Fig. S2). Note also the eye lens-restricted expression of tdTomato originating from the CryaatdTomato transposon.

B) Magnified view of an eye of the double-transgenic calf excited for tdTomato.

C) Control (wildtype) cow eye shown under specific excitation of tdTomato,

C´) Control eye shown under white light illumination.

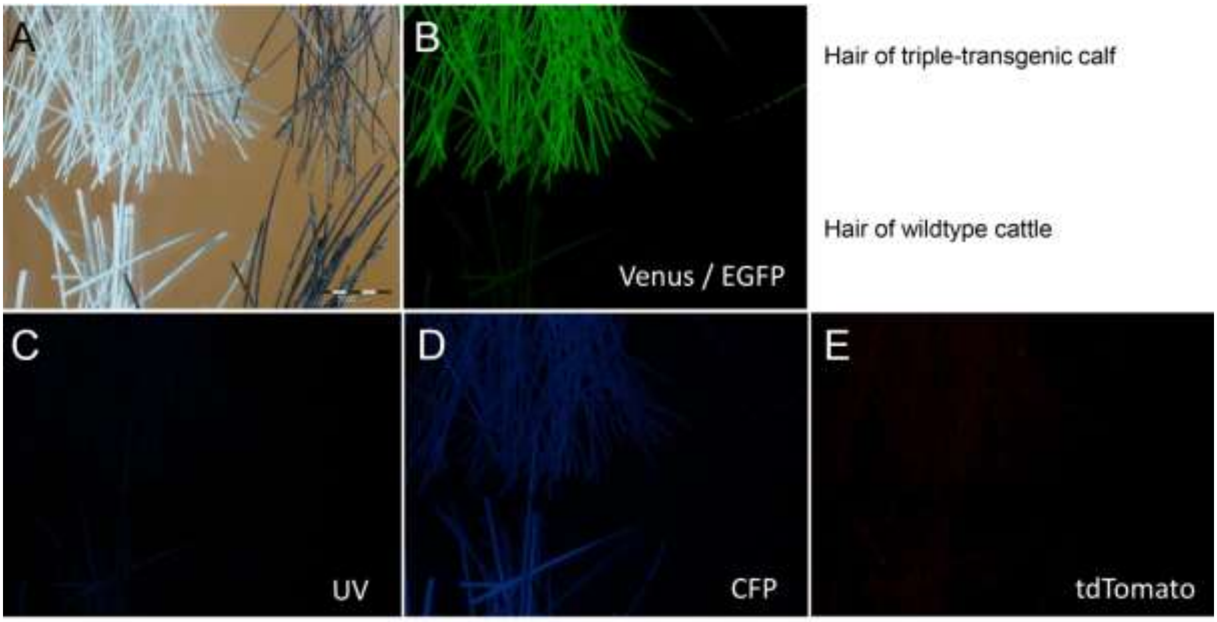

Fig. S2 Deposition of Venus fluorophore in hair of transgenic calf

A) Top: white and black hair of triple-transgenic male calf; bottom: white and black hair of wildtype
B) Same view shown under specific excitation of Venus.
C) Same view shown under specific excitation of a UV fluorophore.
D) Same view shown under specific excitation of a cyan fluorescent protein.
E) Same view shown under specific excitaion of tdTomato.

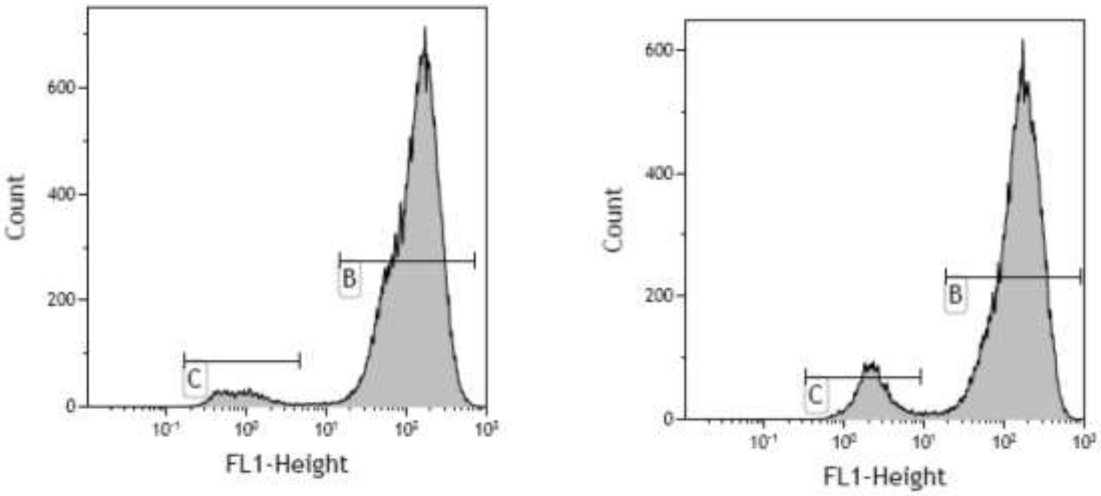

Female 94.5 %

male 88.2 %

Fig. S3 Ration of Venus-positive cells in cultured fibroblasts from the transgenic calves

A) In fibroblasts of the female calf 94.5% of the cells were Venus-positive.

B) In fibroblast cultures of the male calf 88.2 % of the cells were Venus-positive. No expression of the tdTomato could be detected (not shown).

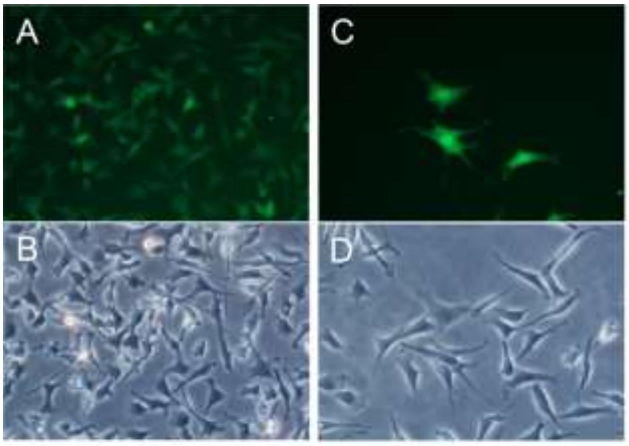

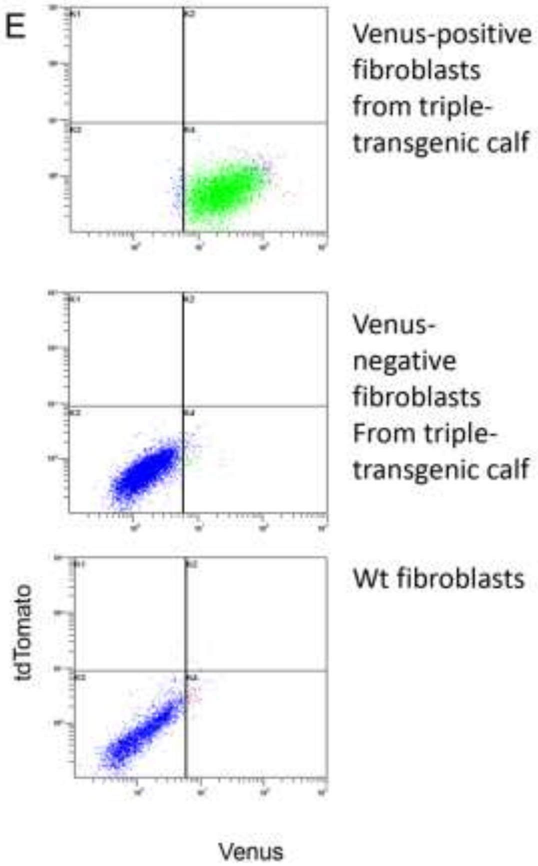

Fig. S4 Cellular mosaicism in cultured fibroblasts from the triple-transgenic calf

A) In fibroblasts of the male calf 88.2 % of the cells were Venus-positive.
B) Corresponding brightfield image (100x mag.)
C) Venus-positive and Venus-negative fibroblasts shown at higher magnification (200x).
D) Corresponding brighfield view.
E) FACS sorted Venus-positive and Venus-negative cell fraction. Note that the tdTomato is not expressed.

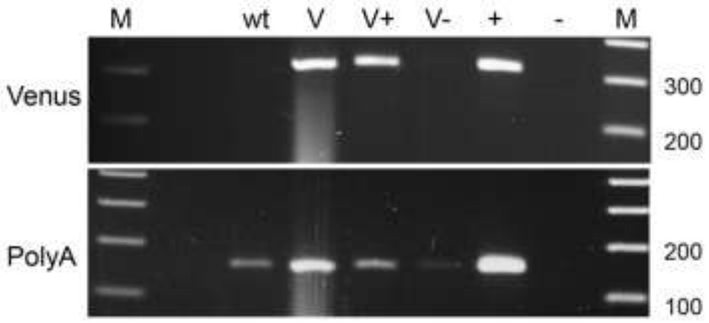

Fig. S5 Genotyping of sorted fibroblasts from double transgenic calf
Top: Venus-specific PCR of wt, wildtype gDNA; V, unsorted fibroblasts of double-transgenic calf; V+, Venus-positive cell fraction; V-, Venus-negative cell fraction; +, positive, and -, negative control.
Bottom: Control PCR with primers for PolyA.
